# Supplementary material for: Genome-Wide Identification and Evolution of the GRF Gene Family and Functional Characterization of PbGRF18 in Pear
Source: Int J Mol Sci. 2023 Sep 28;24(19):14690. doi: 10.3390/ijms241914690 (PMC10572701; doi:10.3390/ijms241914690)
Supplement: Supplementary file 1 [file ijms-24-14690-s001.zip › Figure S3.pdf]

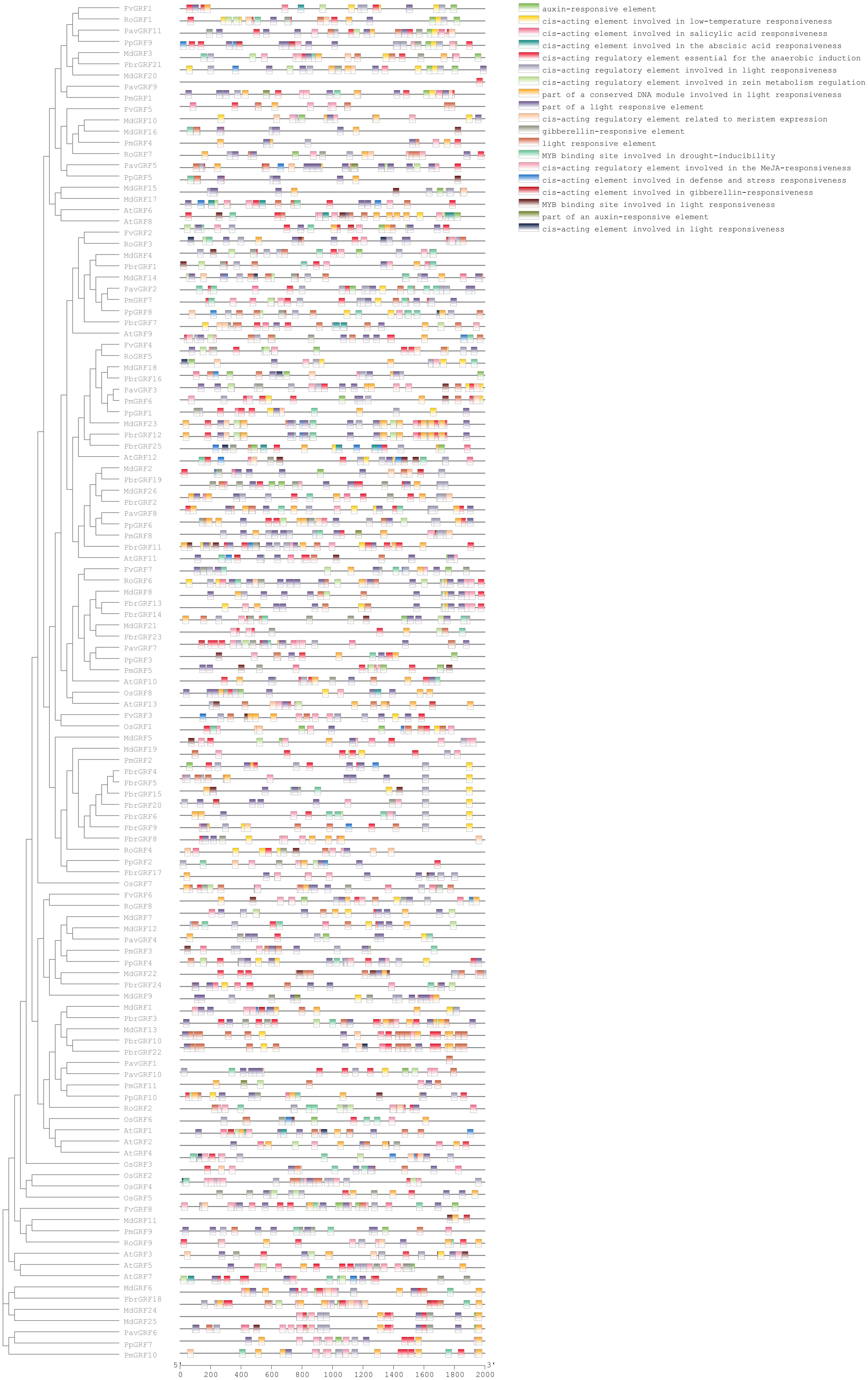
FvGRF1
RoGRF1
PavGRF11
PpGRF9
MdGRF3
PbrGRF21
MdGRF20
PavGRF9
PmGRF1
FvGRF5
MdGRF10
MdGRF16
PmGRF4
RoGRF7
PavGRF5
PpGRF5
MdGRF15
MdGRF17
AtGRF6
AtGRF8
FvGRF2
RoGRF3
MdGRF4
PbrGRF1
MdGRF14
PavGRF2
PmGRF7
PpGRF8
PbrGRF7
AtGRF9
FvGRF4
RoGRF5
MdGRF18
PbrGRF16
PavGRF3
PmGRF6
PpGRF1
MdGRF23
PbrGRF12
PbrGRF25
AtGRF12
MdGRF2
PbrGRF19
MdGRF26
PbrGRF2
PavGRF8
PpGRF6
PmGRF8
PbrGRF11
AtGRF11
FvGRF7
RoGRF6
MdGRF8
PbrGRF13
PbrGRF14
MdGRF21
PbrGRF23
PavGRF7
PpGRF3
PmGRF5
AtGRF10
OsGRF8
AtGRF13
FvGRF3
OsGRF1
MdGRF5
MdGRF19
PmGRF2
PbrGRF4
PbrGRF5
PbrGRF15
PbrGRF20
PbrGRF6
PbrGRF9
PbrGRF8
RoGRF4
PpGRF2
PbrGRF17
OsGRF7
FvGRF6
RoGRF8
MdGRF7
MdGRF12
PavGRF4
PmGRF3
PpGRF4
MdGRF22
PbrGRF24
MdGRF9
MdGRF1
PbrGRF3
MdGRF13
PbrGRF10
PbrGRF22
PavGRF1
PavGRF10
PmGRF11
PpGRF10
RoGRF2
OsGRF6
AtGRF1
AtGRF2
AtGRF4
OsGRF3
OsGRF2
OsGRF4
OsGRF5
FvGRF8
MdGRF11
PmGRF9
RoGRF9
AtGRF3
AtGRF5
AtGRF7
MdGRF6
PbrGRF18
MdGRF24
MdGRF25
PavGRF6
PpGRF7
PmGRF10
5'
3'
0
200
400
600
800
1000
1200
1400
1600
1800
2000
auxin-responsive element
cis-acting element involved in low-temperature responsiveness
cis-acting element involved in salicylic acid responsiveness
cis-acting element involved in the abscisic acid responsiveness
cis-acting regulatory element essential for the anaerobic induction
cis-acting regulatory element involved in light responsiveness
cis-acting regulatory element involved in zein metabolism regulation
part of a conserved DNA module involved in light responsiveness
part of a light responsive element
cis-acting regulatory element related to meristem expression
gibberellin-responsive element
light responsive element
MYB binding site involved in drought-inducibility
cis-acting regulatory element involved in the MeJA-responsiveness
cis-acting element involved in defense and stress responsiveness
cis-acting element involved in gibberellin-responsiveness
MYB binding site involved in light responsiveness
part of an auxin-responsive element
cis-acting element involved in light responsiveness
