# Supplementary material for: Genome-Wide Identification and Evolution of the GRF Gene Family and Functional Characterization of PbGRF18 in Pear
Source: Int J Mol Sci. 2023 Sep 28;24(19):14690. doi: 10.3390/ijms241914690 (PMC10572701; doi:10.3390/ijms241914690)
Supplement: Supplementary file 1 [file ijms-24-14690-s001.zip › Figure S4.pdf]

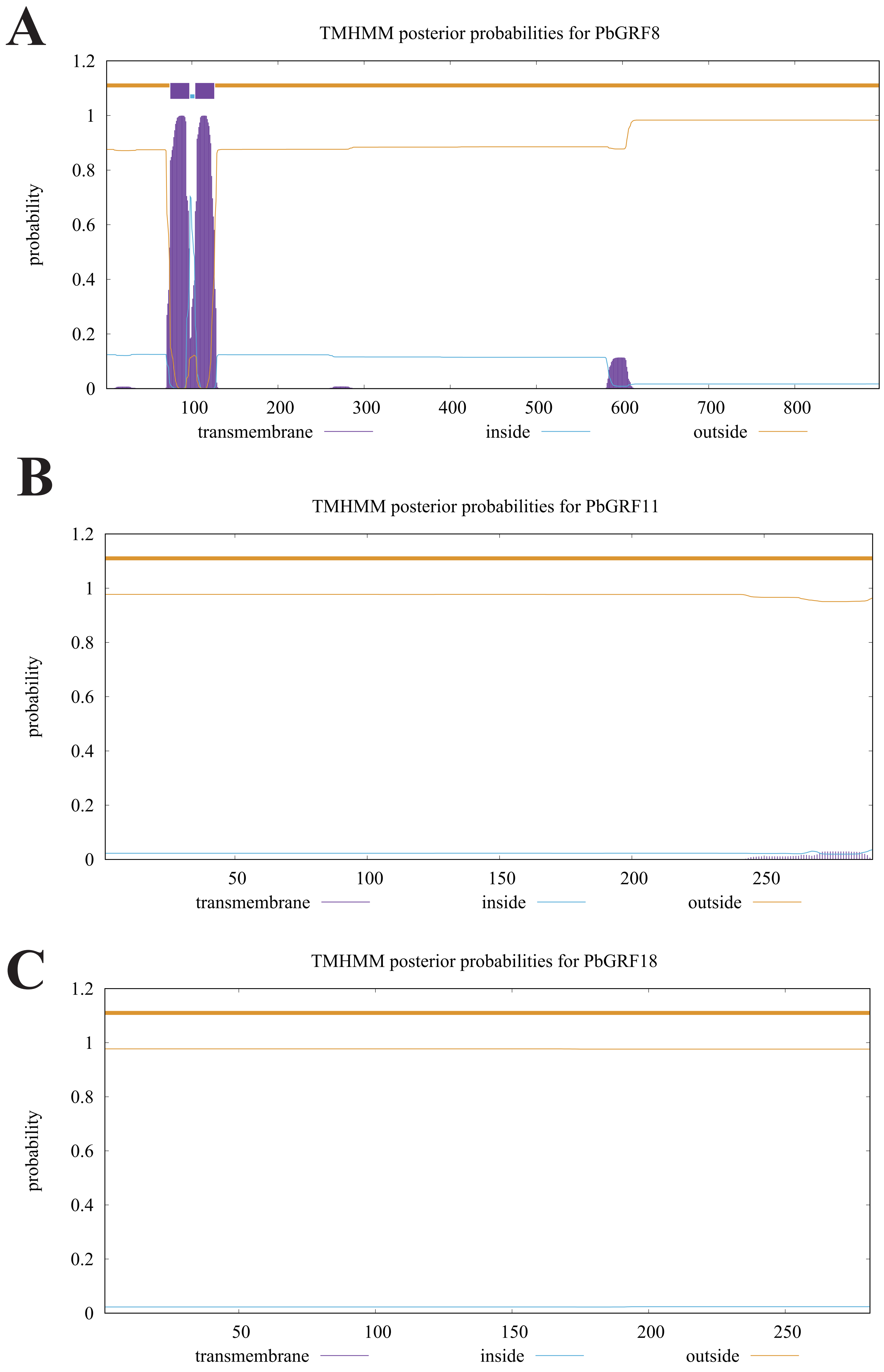
A
TMHMM posterior probabilities for PbGRF8
probability
1.2
1
0.8
0.6
0.4
0.2
0
100
200
300
400
500
600
700
800
transmembrane
inside
outside
B
TMHMM posterior probabilities for PbGRF11
probability
1.2
1
0.8
0.6
0.4
0.2
0
50
100
150
200
250
transmembrane
inside
outside
C
TMHMM posterior probabilities for PbGRF18
probability
1.2
1
0.8
0.6
0.4
0.2
0
50
100
150
200
250
transmembrane
inside
outside
