# Supplementary material for: Genome-Wide Identification and Evolution of the GRF Gene Family and Functional Characterization of PbGRF18 in Pear
Source: Int J Mol Sci. 2023 Sep 28;24(19):14690. doi: 10.3390/ijms241914690 (PMC10572701; doi:10.3390/ijms241914690)
Supplement: Supplementary file 1 [file ijms-24-14690-s001.zip › supplementary caption.pdf]

Figure S1. A bar graph showing the numbers of acidic and basic members for the PbGRF, AtGRF, OsGRF, FvGRF, PavGRF, PmGRF, RoGRF, PpGRF, and MdGRF proteins.

Figure S2. The motif prediction for the GRF proteins, motifs 1-10 ranging in length from 15 to 43 amino acids.

Figure S3. The CREs distribution in the promoter regions of the GRF genes, a total of 19 CREs were identified.

Figure S4. Prediction of PbGRFs transmembrane structural domains. A: The prediction of PbGRF8 transmembrane structural domains. B: Prediction of PbGRF11 transmembrane structural domains. C: Prediction of PbGRF18 transmembrane structural domains

Table S1. The basic informations of 26 MdGRFs, 8 FvGRFs, 11 PmGRFs, 10 PpGRFs, 21 PcGRFs, 9 RoGRFs, 11 PavGRFs, 8 OsGRFs, and 13AtGRFs.

Table S2. WGD/segmental duplication analysis of *PbGRFs*.

Table S3. The distribution and types of CREs in the *PbGRF* promoters.

Table S4. The RPKM of *PbGRFs* in seven different tissues of ‘dangshansuli’.

Table S5. The RPKM of *PbGRFs* in six developmental stages of ‘Dangshansuli’ pear fruits.

Table S6. The primers used in this study.
