# Supplementary figures and images for: Genome-Wide Identification and Evolution of the GRF Gene Family and Functional Characterization of PbGRF18 in Pear
Source: Int J Mol Sci. 2023 Sep 28;24(19):14690. doi: 10.3390/ijms241914690 (PMC10572701; doi:10.3390/ijms241914690)

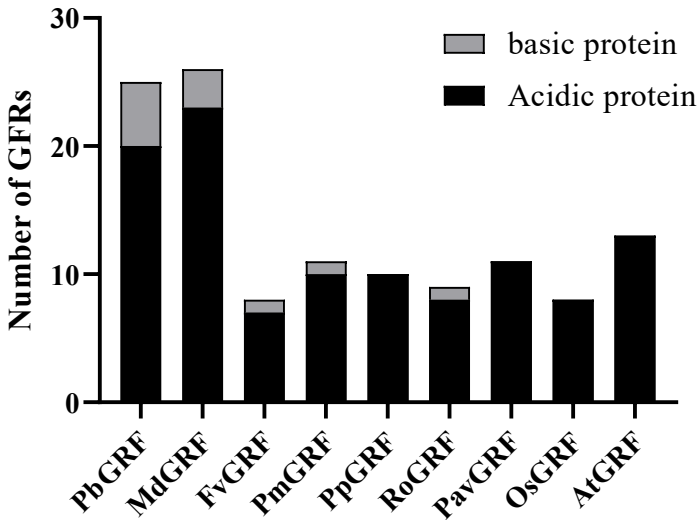

Supplement: Supplementary file 1 [file ijms-24-14690-s001.zip › Figure S1.pdf]

Motif 1

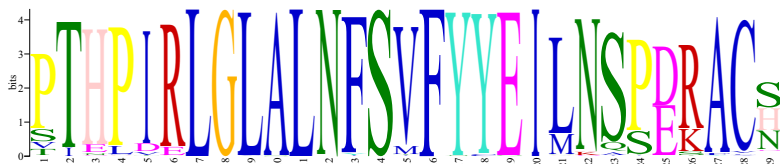

Motif 2

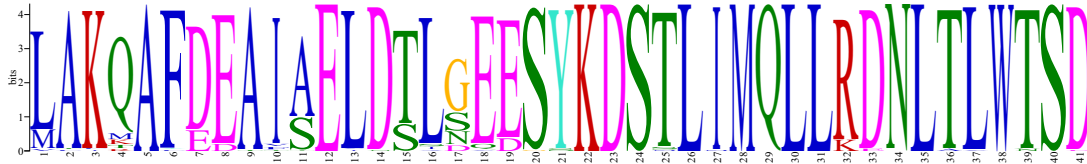

Motif 3

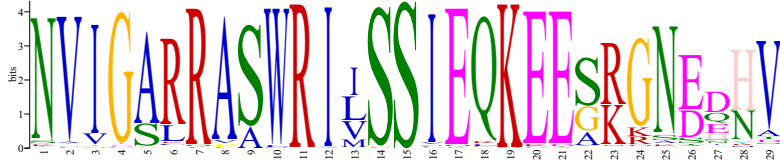

Motif 4

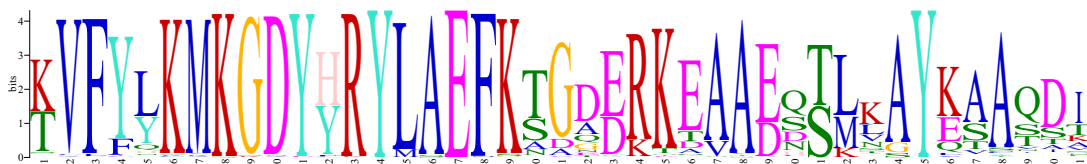

Motif 5

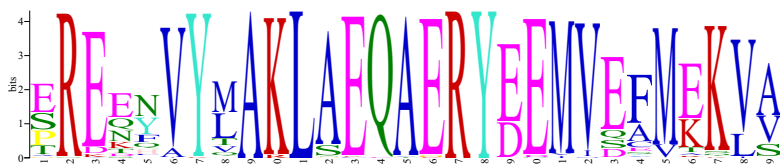

Motif 6

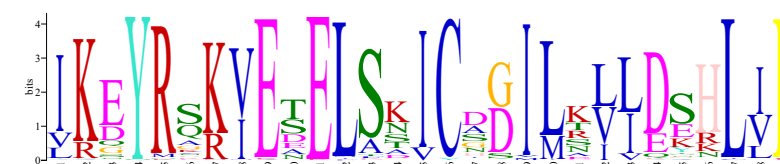

Motif 7

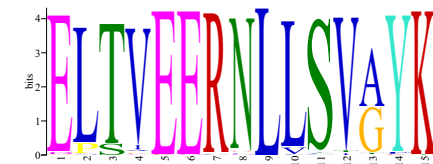

Motif 8

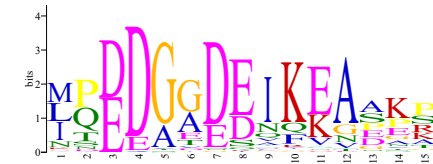

Motif 9

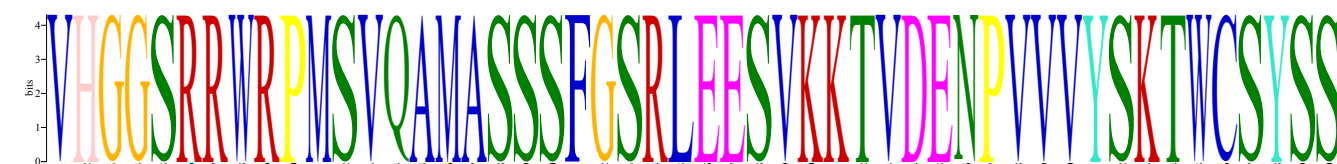

Motif 10

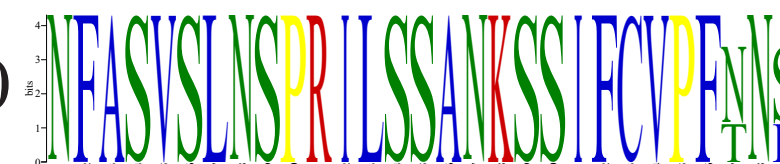

Supplement: Supplementary file 1 [file ijms-24-14690-s001.zip › Figure S2.pdf]
